# Bacteria Contribute to Plant Secondary Compound Degradation in a Generalist Herbivore System

**DOI:** 10.1128/mBio.02146-20

**Published:** 2020-09-15

**Authors:** Charlotte B. Francoeur, Lily Khadempour, Rolando D. Moreira-Soto, Kirsten Gotting, Adam J. Book, Adrián A. Pinto-Tomás, Ken Keefover-Ring, Cameron R. Currie

**Affiliations:** a Department of Bacteriology, University of Wisconsin—Madison, Madison, Wisconsin, USA; b Department of Energy Great Lakes Bioenergy Research Center, University of Wisconsin—Madison, Madison, Wisconsin, USA; c Sección de Entomología Medica, Departamento de Parasitología, Facultad de Microbiología, Universidad de Costa Rica, San José, Costa Rica; d Laboratory of Genetics, University of Wisconsin—Madison, Madison, Wisconsin, USA; e Centro de Investigación en Estructuras Microscópicas, Universidad de Costa Rica, San José, Costa Rica; f Departamento de Bioquímica, Facultad de Medicina, Universidad de Costa Rica, San José, Costa Rica; g Centro de Investigación en Biología Celular y Molecular, Universidad de Costa Rica, San José, Costa Rica; h Departments of Botany and Geography, University of Wisconsin—Madison, Madison, Wisconsin, USA; University of Hawaii at Manoa

**Keywords:** attine, detoxification, fungus garden, leaf-cutter ant, symbiosis

## Abstract

Herbivores must overcome a variety of plant defenses, including coping with plant secondary compounds (PSCs). To help detoxify these defensive chemicals, several insect herbivores are known to harbor gut microbiota with the metabolic capacity to degrade PSCs. Leaf-cutter ants are generalist herbivores, obtaining sustenance from specialized fungus gardens that act as external digestive systems and which degrade the diverse collection of plants foraged by the ants. There is *in vitro* evidence that certain PSCs harm Leucoagaricus gongylophorus, the fungal cultivar of leaf-cutter ants, suggesting a role for the *Proteobacteria*-dominant bacterial community present within fungus gardens. In this study, we investigated the ability of symbiotic bacteria present within fungus gardens of leaf-cutter ants to degrade PSCs. We cultured fungus garden bacteria, sequenced the genomes of 42 isolates, and identified genes involved in PSC degradation, including genes encoding cytochrome P450 enzymes and genes in geraniol, cumate, cinnamate, and α-pinene/limonene degradation pathways. Using metatranscriptomic analysis, we showed that some of these degradation genes are expressed *in situ*. Most of the bacterial isolates grew unhindered in the presence of PSCs and, using gas chromatography-mass spectrometry (GC-MS), we determined that isolates from the genera *Bacillus*, *Burkholderia*, *Enterobacter*, *Klebsiella*, and *Pseudomonas* degrade α-pinene, β-caryophyllene, or linalool. Using a headspace sampler, we show that subcolonies of fungus gardens reduced α-pinene and linalool over a 36-h period, while *L. gongylophorus* strains alone reduced only linalool. Overall, our results reveal that the bacterial communities in fungus gardens play a pivotal role in alleviating the effect of PSCs on the leaf-cutter ant system.

## Abstract (precis)

Leaf-cutter ants are dominant neotropical herbivores capable of deriving energy from a wide range of plant substrates. The success of leaf-cutter ants is largely due to their external gut, composed of key microbial symbionts, specifically, the fungal mutualist *L. gongylophorus* and a consistent bacterial community. Both symbionts are known to have critical roles in extracting energy from plant material, yet comparatively little is known about their roles in the detoxification of plant secondary compounds. In this study, we assessed if the bacterial communities associated with leaf-cutter ant fungus gardens can degrade harmful plant chemicals. We identify plant secondary compound detoxification in leaf-cutter ant gardens as a process that depends on the degradative potential of both the bacterial community and *L. gongylophorus*. Our findings suggest that the fungus garden and its associated microbial community influence the generalist foraging abilities of the ants, underscoring the importance of microbial symbionts in plant substrate suitability for herbivores.

## INTRODUCTION

Plants defend themselves from herbivores through the production of an extraordinarily diverse set of plant secondary compounds (PSCs) (1–4). Not only are these chemicals diverse in structure and toxicity, but also the mechanisms behind their synthesis, storage, and release are complex, depending on the type of damage, the taxon inflicting the damage, the species of plant, environmental conditions (e.g., hours of sunlight, temperature, and moisture), and other factors (3, 5, 6). The range of plant chemical defenses requires herbivorous insects to engage in multiple strategies to counter toxic effects of PSCs, including harboring microbial symbionts that aid in detoxification. Microbes associated with mountain pine beetles (7–9), red turpentine beetles (8), pine weevils (10), gypsy moths (11), apple maggot flies (12), termites (13), and coffee berry borers (14) have been found to play an important role in PSC degradation. Understanding the role of microbial symbionts in PSC detoxification is critical, since the capacity of insects to mitigate PSC toxicity is an important factor in determining host plant range (15).

**FIG 1:**
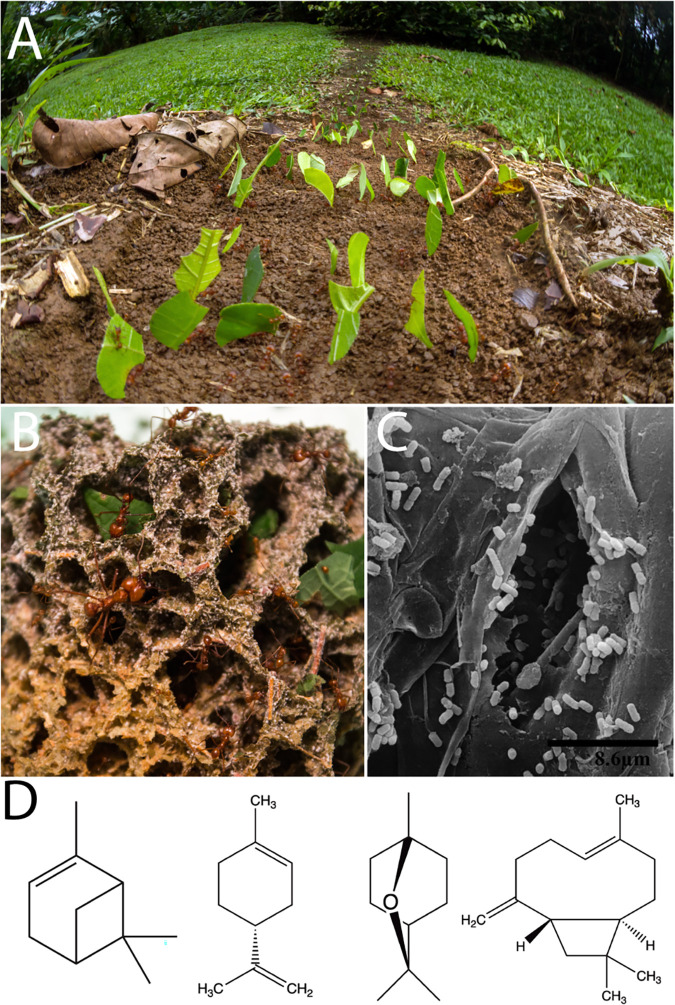
Leaf-cutter ants are dominant herbivores through the formation of multiple microbial symbioses. Leaf-cutter ants (*Atta cephalotes*) form visible foraging trails (A) as they cut fresh leaf material at large scales as a food source for their specialized fungus gardens (B). Bacteria are known to coexist within the fungus garden (C), and PSCs can be detected from the fresh leaf material in the fungus gardens using GC-MS. One *Atta laevigata* sample collected in an area with eucalyptus had detectable amounts of PSCs, such as α-pinene, *p*-cymene, eucalyptol, and caryophyllene oxide (32) (D). Photo credits: panel A, Alexander Wild; panel B, Lily Khadempour; panel C, Rolando Moreira-Soto, reprinted from reference 26.

Leaf-cutter ants, two genera within the monophyletic fungus-growing ant subtribe, are dominant herbivores in most Neotropical ecosystems and are able to forage from a diverse array of plants. In a long-term study, active colonies of two species of leaf-cutter ants, Atta colombica and Atta cephalotes, were observed cutting leaves from 67 to 77% of all plant species (86 and 59 plant species, respectively) recorded in their respective foraging areas (16). In a year-long study by Wirth and colleagues (17), one colony of A. colombica foraged from the leaves or flowers of 126 species, representing 91 genera and 52 families. In mature colonies of *Atta*, the ants forage ravenously, forming massive foraging columns that create distinctive trails (Fig. 1A). The leaf-cutter ants do not directly consume the plant substrate but rather use it to feed Leucoagaricus gongylophorus (Agaricales: Agaricaceae), their obligate fungal mutualist. In return, L. gongylophorus degrades the leaf substrate and serves as food for the ants, providing energy and nutrients in the form of specialized hyphal swellings known as gongylidia (18, 19). This process occurs in structures known as fungus gardens (Fig. 1B), which are maintained in underground chambers. Other lineages of fungus-farming ants bring nonleaf substrate to *Leucoagaricus* spp. and contain 1 to 20 fungus garden chambers with a total colony size of no more than a few thousand workers (20, 21). In contrast, mature colonies of the leaf-cutting ant genus *Atta* can be composed of hundreds of fungus garden chambers, which provide nutrition to millions of larvae, pupae, and emerged workers (19, 22).

The fungus garden, which functionally serves as the ants’ external gut, includes the main fungal symbiont *L. gongylophorus* and a diverse and abundant community of bacteria. In contrast, the internal gut of leaf-cutter ants has a reduced bacterial community, with adult worker guts associated with *Wolbachia* or *Mollicutes* (23–25). Culturing, scanning electron microscopy (26) (Fig. 1C), and metagenomics of fungus gardens demonstrate the consistent presence of garden bacteria and have established a core bacterial community that consists mostly of *Proteobacteria*, the majority being in the class *Gammaproteobacteria* (27–32). Metagenomic studies show evidence for potential roles of garden bacteria in amino acid, vitamin, iron, and terpenoid nutrient supplementation, among other processes (27, 32). However, metagenomic predictions cannot establish these roles definitively, as they only demonstrate that garden bacteria have the genetic potential to carry out these functions. Attempts have been made to use proteomics (33) and transcriptomics (34) to examine the bacterial community *in situ*; however, this has not proven fruitful due to the low ratio of bacterial to fungal biomass in fungus gardens. Fortunately, easily culturable bacterial genera, such as *Burkholderia*, *Enterobacter*, *Klebsiella*, *Pantoea*, and *Pseudomonas*, are known to be consistently present in fungus gardens. The advantage of having cultured isolates was leveraged in a previous study with *Klebsiella* and *Pantoea*, demonstrating the critical role of these two genera in fixing nitrogen that assimilates in the bodies of the leaf-cutter ants (35).

In this study, we examined the ability of garden bacteria from leaf-cutter ants to metabolize PSCs. We focus on bacteria isolated from fungus gardens of fungus-growing ants to investigate the potential of garden bacteria to tolerate and degrade PSCs. First, we used previously isolated strains of *L. gongylophorus* and *Leucoagaricus* sp. WM170124-07 and determined their susceptibility to eight PSCs that were selected based on foraging and leaf extract studies (17, 25, 36–44). Next, we exposed isolates of garden bacteria to the same eight PSCs to determine their susceptibility and sequenced the genomes of 42 isolates of garden bacteria and predicted the presence of genes involved in PSC degradation. Then, using gas chromatography-mass spectrometry (GC-MS), we quantified the *in vitro* ability of 15 isolates of garden bacteria to degrade four PSCs. Finally, we measured reduction of two PSCs by fungus gardens from our laboratory colonies of *Atta cephalotes* using a headspace sampler coupled to a gas chromatograph. For additional evidence, we analyzed previously generated metagenomes of garden bacteria, genomes of *Leucoagaricus* spp., and metatranscriptomes to investigate the presence and expression of genes involved in PSC degradation.

## RESULTS

### *L. gongylophorus* and *Leucoagaricus* spp. are susceptible to PSCs and are not predicted to carry genes involved in PSC degradation.

**FIG 2:**
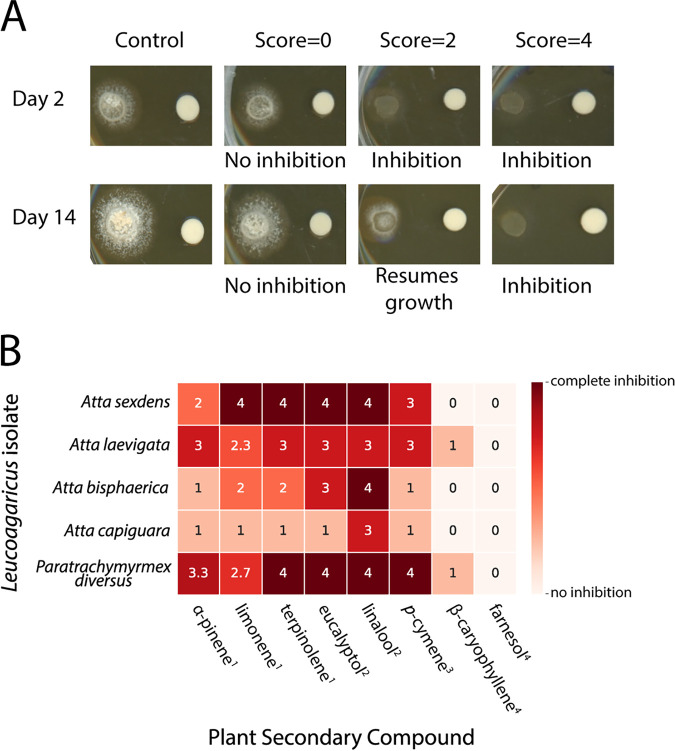
*L. gongylophorus* and *Leucoagaricus* sp. strains have variable tolerances of different PSCs. The growth of *L. gongylophorus* and *Leucoagaricus* sp. WM170124-07 was qualitatively scaled (A). Each isolate was exposed to eight PSCs using a disc assay. Growth was scored for each isolate and averaged across the three technical replicates (B). The terpene classes are indicated by superscript numbers as follows: 1, monoterpene; 2, terpenoid; 3, alkylbenzene related to monoterpene; and 4, sesquiterpene.

We tested four different strains of *L. gongylophorus* and *Leucoagaricus* sp. WM170124-07 for the ability to grow in the presence of eight different PSCs (Fig. 2). The chosen PSCs were selected because they both are commercially available in purified form and are either detected in leaf-cutter ant fungus gardens (32) or predicted to be encountered by leaf-cutter ants based on multiple foraging and chemical profile studies (17, 25, 36–44). *Leucoagaricus* sp. WM170124-07 from a Paratrachymyrmex diversus colony was the most generally inhibited, with complete growth inhibition from terpinolene, eucalyptol, linalool, and *p*-cymene and high inhibition from α-pinene and limonene. *Leucoagaricus* sp. WM170124-07 from a *P. diversus* colony and *L. gongylophorus* from an Atta laevigata colony were the only fungal cultivars that were inhibited by the sesquiterpene β-caryophyllene. *L. gongylophorus* from an Atta sexdens colony exhibited high sensitivity to PSCs, with complete inhibition from limonene, terpinolene, eucalyptol, and linalool. *L. gongylophorus* from an Atta capiguara colony was the most resistant to the PSCs tested, exhibiting high inhibition only in the presence of linalool while exhibiting low to no inhibition in the presence of the remaining seven compounds (Fig. 2B).

**FIG 3:**
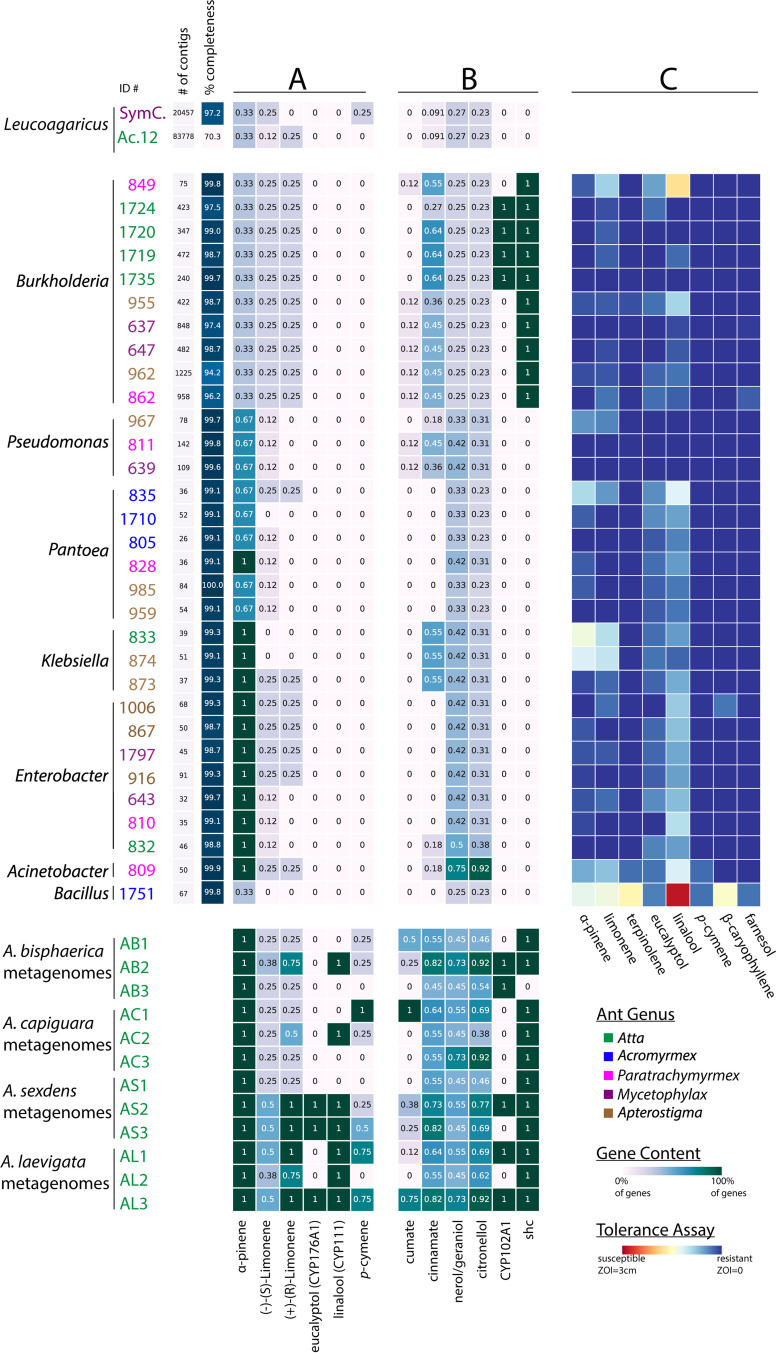
Fungal cultivars, fungus garden bacteria, and fungus garden bacterial metagenomes contain different proportions of PSC degradation genes, and fungus garden bacteria can tolerate PSCs. *Leucoagaricus gongylophorus*/*Leucoagaricus* spp. strains, fungus garden bacteria, and fungus garden bacterial metagenomes are color-coded by ant colony type. Genome quality statistics (number of contigs and BUSCO percent completeness metric) are provided directly next to the bacterial isolates, as well as at 10.6084/m9.figshare.12747092. (A) Genes and pathways involved in the degradation of PSCs used in *in vitro* and *in vivo* experiments within this study, specifically, α-pinene (three genes), (−)-(*S*)-limonene (eight genes), and (+)-(*R*)-limonene degradation (four genes) (KEGG map00903) and eucalyptol (CYP176A1), linalool (CYP111), and *p-*cymene (four genes) (map01220:M00419). (B) Genes and pathways involved in the degradation of other PSCs, not used in any *in vitro* or *in vivo* experiments within this study, specifically, cumate (eight genes) (map01220:M00539), *trans*-cinnamate (11 genes) (map01220:M00545), and nerol, geraniol, and citronellol (map00281). Twelve KEGG orthologs (KOs) are known in the nerol/geraniol degradation route, and 13 KOs are involved in citronellol degradation. All of the pathways are presented as proportions with 1 being 100% of the genes predicted to be present and 0 being 0% of the genes predicted to be present. CYP176A1, CYP111, CYP102A1, and *shc* are single genes, so “0” or “1” indicates the absence or presence of that sequence, respectively. A total of 31/42 annotations are displayed here. The annotations for the remaining 11 genomes are included in (10.6084/m9.figshare.12746852 and 10.6084/m9.figshare.12746861). (C) 31 isolates with whole-genome sequences were tested for tolerance of eight PSCs. Data from a larger set of 46 isolates (15 without whole-genome sequences) are provided at 10.6084/m9.figshare.12746990.

Based on the inhibition profiles, we hypothesized that *L. gongylophorus* would not be predicted to contain genes involved in PSC degradation. To assess genomic PSC degradation potential, we conducted BLAST and Kyoto Encyclopedia of Genes and Genomes (KEGG)-based annotations on two existing fungal cultivar genomes. We investigated the presence of known genes involved in α-pinene, limonene, eucalyptol, linalool, and *p-*cymene degradation (Fig. 3A; see also 10.6084/m9.figshare.12746852 and 10.6084/m9.figshare.12746861). In addition, we investigated other genes involved in degradation of monoterpenes and aromatic compounds (Fig. 3B; 10.6084/m9.figshare.12746852 and 10.6084/m9.figshare.12746861). Importantly, all PSC degradation pathways except *p-*cymene and cumate are incomplete, with known transformation reactions lacking respective enzyme information in KEGG (10.6084/m9.figshare.12746864, 10.6084/m9.figshare.12746867, 10.6084/m9.figshare.12746870, and 10.6084/m9.figshare.12746873). Therefore, gene content does not necessarily reflect the functionality of these pathways. Overall, the two genomes, one from *A. cephalotes* (*L. gongylophorus* [Ac12]) and one from Cyphomyrmex costatus (*Leucoagaricus* sp. [SymC.cos]), lacked most of the gene sets analyzed (Fig. 3A; 10.6084/m9.figshare.12746852 and 10.6084/m9.figshare.12746861), suggesting that the fungal cultivar may depend on fungus garden bacteria for degradation of these compounds.

### Fungus garden bacteria can tolerate PSCs and are predicted to carry genes involved in PSC degradation.

We assessed the ability of 46 fungus garden bacterial isolates to grow in the presence of eight PSCs. Most bacterial isolates were able to grow uninhibited by the PSCs (Fig. 3C; 10.6084/m9.figshare.12746990). Linalool was the most inhibitory against the bacterial isolates, causing some degree of inhibition against all isolates except *Pseudomonas* and three *Burkholderia* isolates. Farnesol, β-caryophyllene, and terpinolene did not inhibit the majority of bacterial isolates, causing small zones of inhibition in the *Bacillus* isolate as well as one to two other isolates in the genera *Burkholderia* and *Pantoea*. α-Pinene and limonene caused slightly more inhibition, especially in *Bacillus* and two *Klebsiella* isolates, but most bacteria were resistant or only slightly susceptible to these two compounds.

To assess the potential of garden bacteria to degrade PSCs, we conducted BLAST- and KEGG-based annotations on 42 whole genomes of bacteria isolated from fungus gardens and 12 garden bacterial metagenomes (32) (Fig. 3A and B). The analyses were identical to those performed with the two fungal cultivar genomes, described above. In contrast to the fungal genomes, fungus garden bacterial isolates and metagenomes were predicted to encode higher proportions of genes involved in PSC degradation.

**(i) α-Pinene and limonene degradation genes.** Many of the enzymes in the α-pinene/limonene KEGG degradation pathway are uncharacterized (10.6084/m9.figshare.12746864). For α-pinene degradation, three enzymes are known to be involved in the transformation of *cis*- or *trans*-2-methyl-5-isopropylhexa-2,5,-dienoyl-coenzyme A to 3-hydroxy-2,6,dimethyl-5-methylene-heptanoyl-coenzyme A in the later stages of one of the degradation routes (K01692, K01825, and K01782). Specifically, Acinetobacter, *Enterobacter*, *Klebsiella*, and one *Pantoea* isolate are predicted to encode all three (Fig. 3A). The remaining bacterial isolates in the genera *Bacillus*, *Burkholderia*, *Pantoea*, and *Pseudomonas* had at least one out of three shared genes (10.6084/m9.figshare.12746861). All of the metagenome samples were predicted to contain all three genes related to these enzymes.

There are eight characterized enzymes associated with (−)-(*S*)-limonene degradation (10.6084/m9.figshare.12746864). No isolates surveyed were predicted to encode the enzymes involved in the first steps of (−)-(*S*)-limonene degradation (i.e., limonene monooxygenases K07381, K07382, and K14733), indicating that is unlikely any of these isolates have fully functional (−)-(*S*)-limonene degradation capabilities. However, all Acinetobacter, *Burkholderia*, and *Paraburkholderia* isolates were predicted to contain the gene encoding monoterpene epsilon-lactone hydrolase (K14731), which is involved the intermediate/terminal steps of monocyclic monoterpene degradation (45). Across *Enterobacter*, *Klebsiella*, and *Pantoea* isolates, only a subset were predicted to encode K14731 (4/7 *Enterobacter* isolates, 1/4 *Klebsiella* isolates, and 1/10 *Pantoea i*solates). In the metagenomes surveyed, 25 to 50% of the eight genes involved in (−)-(*S*)-limonene degradation were present (Fig. 3A).

There are two degradation routes for (+)-(*R*)-limonene (10.6084/m9.figshare.12746864). The initial enzyme necessary to transform (+)-(*R*)-limonene to (*+*)-*trans*-carveol is currently uncharacterized; however, one *Paraburkholderia* isolate was found to possess the gene for the enzyme involved in the second step converting (+)-*trans*-carveol to (4*S*)-carvone (K12466). None of the bacterial isolates were predicted to carry genes for the second degradation route (Fig. 3A). Metagenomes contained a range of 25 to 100% of the four genes involved in (+)-(*R*)-limonene degradation (Fig. 3A).

**(ii) Eucalyptol and linalool degradation.** The cytochrome P450s 8-cineole 2-endo-monooxygenase (CYP176A1) and linalool 8-monooxygenase (CYP111) are known to be involved in the oxidation of eucalyptol and linalool, respectively. Genes for these enzymes were not identified in the bacterial isolate genomes surveyed. However, fungus garden bacterial metagenomes from three colonies (two *A. sexdens* and one *A. laevigata*) are predicted to encode CYP176A1, and seven colonies (one Atta bisphaerica, one *A. capiguara*, two *A. sexdens*, and three *A. laevigata*) are predicted to encode CYP111 (Fig. 3A; 10.6084/m9.figshare.12746852 and 10.6084/m9.figshare.12746861).

**(iii) *p*-Cymene and cumate degradation.** The *p*-cymene pathway is fully characterized, with four enzymes involved in the transformation of *p*-cymene into cumate, which is further degraded via the well-characterized cumate pathway to *cis*-2-hydroxy-penta-2,4-dienoate (10.6084/m9.figshare.12746867). None of the isolates were predicted to carry genes involved in *p*-cymene degradation. However, the metagenome annotation did predict *p-*cymene degradation genes, with one *A. capiguara* metagenome (AC1) including all four genes for a complete *p*-cymene degradation pathway (Fig. 3A; 10.6084/m9.figshare.12746861). Ten of the isolates contained one gene from the cumate pathway, with seven *Burkholderia* and two *Pseudomonas* isolates predicted to contain genes encoding different components of the *p*-cumate 2,3-dioxygenase enzyme (K16303 or K16304) and one *Pantoea* isolate predicted to have a gene encoding 2,3-dihydroxy-*p*-cumate/2,3-dihydroxybenzoate 3,4-dioxygenase (K10621). Three metagenomes (one *A. bisphaerica*, one *A. capiguara*, and one *A. laevigata*) were predicted to include 50% or more of the genes in the cumate pathway, with one sample (AC1) predicted to encode the entire cumate degradation pathway.

**(iv) Other PSC degradation pathways and associated genes.** Pathways for β-caryophyllene, farnesol, and terpinolene degradation are not known, so we could not assess bacterial *in silico* potential to degrade these compounds. However, other PSC degradation pathways are described, including for *trans*-cinnamate (Fig. 3B; 10.6084/m9.figshare.12746870), nerol, geraniol, citronellol (Fig. 3B; 10.6084/m9.figshare.12746873), and diterpenes (10.6084/m9.figshare.12746852). In addition, we analyzed the presence of a set of cytochrome P450s and individual genes *saxA* and *shc* (10.6084/m9.figshare.12746852 and 10.6084/m9.figshare.12746861).

Finally, to confirm that our bacterial isolates are representative of the community in leaf-cutter ant fungus gardens, we created whole-genome phylogenies with fungus garden bacterial isolates in the classes *Betaproteobacteria* and *Gammaproteobacteria* from the current study and previous studies and with closely related bacteria (10.6084/m9.figshare.12746999). While fungus garden bacteria vary widely at the species and strain levels, we confirmed that the strains we isolated are representative of the abundant genera in fungus gardens.

### Fungus garden metatranscriptomes indicate bacterial expression of PSC degradation genes.

Previously generated metatranscriptomes of fungus gardens (34) were analyzed for *in situ* expression of PSC degradation genes. We detected the expression of α-pinene, limonene, citronellol, nerol, and geraniol degradation genes in the metatranscriptomes of three leaf-cutter ant colonies: two *A. cephalotes* (FG1 and FG2) and one *A. colombica* (FG3) (10.6084/m9.figshare.12747113 and 10.6084/m9.figshare.12747032). Specifically, we detected the expression of six genes involved in α-pinene and limonene degradation in the range of 0.1681 to 3,724 transcripts per million (TPM), including expression of the enzyme responsible for initial transformation of limonene, limonene 1,2-monooxygenase (K14733), at 0.5063 TPM in FG1. In addition, we detected 11 genes involved in nerol, citronellol, and geraniol degradation in the range of 0.02535 to 27.18 TPM, including geraniol dehydrogenase (K12957) at 3.776 TPM in FG1 and 0.02535 TPM in FG2 and citronellol/citronellal dehydrogenase (K13774) at 0.1472 TPM in FG1. For comparison, housekeeping gene (*gyrB*, *rpoB*, *rpoD*, and *rpsL*) expression levels were detected in the range of 0.1818 to 28.92 TPM. Overall, these results indicate that fungus garden bacteria express PSC degradation genes in the environment.

### Fungus garden bacteria are able to degrade PSCs *in vitro*.

**FIG 4:**
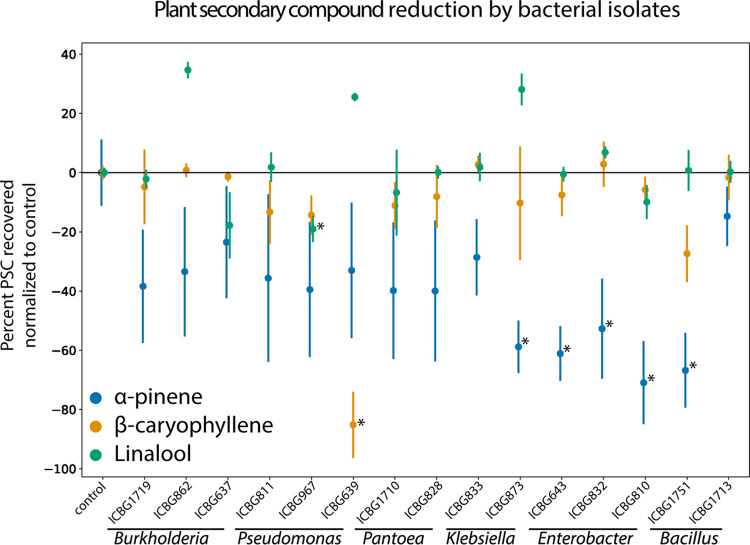
PSC reduction *in vitro* by 15 bacterial isolates. Bacterial isolates are grouped by their genus-level identification on the *x* axis. The *y* axis is the percent change of PSC recovered compared to a nonbacterial control vial (10% TSB plus compound). Each point represents the average of three GC-MS measurements, and the bars show the standard deviations of the observations. One-sample Student’s *t* tests were used to assess reduction of PSCs by bacterial isolates using a Bonferroni-corrected *P* value threshold of 0.0033.

All *Enterobacter* isolates (ICBG810, *P* = 0.0004; ICBG643, *P* = 0.0018; and ICBG832, *P* = 0.0062) significantly reduced α-pinene (*t* test, Bonferroni correction: α = 0.0033) during exponential growth (Fig. 4). In addition, one of two *Klebsiella* isolates (ICBG873, *P* = 0.0026) and one of two *Bacillus* isolates (ICBG1751, *P* = 0.0008) significantly reduced α-pinene. Other bacterial isolates showed various ranges of reduction of α-pinene between vials, resulting in large variability and lack of significance with the Bonferroni-corrected α value. No isolates significantly reduced α-pinene within stationary growth (10.6084/m9.figshare.12747011). One of three *Pseudomonas* isolates (ICBG639, *P* < 0.0001) significantly reduced β-caryophyllene in the exponential environment. The same *Pseudomonas* isolate (ICBG639, *P* = 0.0014) also significantly reduced β-caryophyllene in the stationary phase. Linalool was reduced by two isolates, one each of *Burkholderia* (ICBG637, exponential *P* = 0.0036 and stationary *P* = 0.0008) and *Pseudomonas* (ICBG967, exponential *P* = 0.0020 and stationary *P* = 0.0024). Finally, eucalyptol, the fourth compound tested, was not reduced by any of the isolates tested (10.6084/m9.figshare.12747017). In all cases, no breakdown products of the tested PSCs were detected by GC-MS.

### Fungus garden subcolonies reduce α-pinene and linalool, while *L. gongylophorus* isolates reduce only linalool.

**FIG 5:**
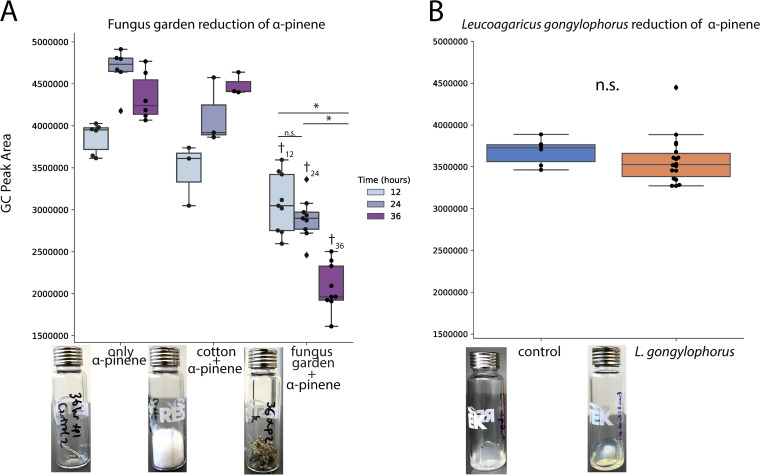
Measurement of α-pinene in the headspace of fungus gardens and *L. gongylophorus* over time. Three subcolonies of three *Atta cephalotes* colonies were exposed to α-pinene for 12, 24, and 36 h and reduction of α-pinene levels was measured in the headspace (A). *L. gongylophorus* cultured from laboratory colonies, as well as the *L. gongylophorus* strains tested for Fig. 2, were cultured on PDA and then exposed to α-pinene for 36 h. Headspace measurements were collected and compared to that of a vial with only PDA (B). In both panels, each point represents a single measurement and is superimposed on top of a boxplot. The *y* axis indicates the area under the curve corresponding to the level of α-pinene in the headspace of each vial. The asterisks indicate significance (*P* < 0.05) between the fungus garden samples at different time points. The dagger indicates significance (*P* < 0.05) between the fungus garden sample and the control vials at the same time point. In the *A. cephalotes* headspace experiments (A), a linear-mixed regression model with time and treatment as fixed effects and ant colony as random effects was used to assess α-pinene headspace levels (α = 0.05). To compare signal peaks of vials containing *L. gongylophorus* and control vials (B), a Welch two-sample *t* test (α = 0.05) was performed. The photographs show examples of the experimental setup for each treatment.

Subcolonies from three *A. cephalotes* colonies were exposed to α-pinene (Fig. 5A). There was significant reduction of α-pinene in the fungus garden samples at 12 h (only α-pinene versus fungus garden plus α-pinene, *P* < 0.0001; cotton plus α-pinene versus fungus garden plus α-pinene, *P* = 0.1191), 24 h (only α-pinene versus fungus garden plus α-pinene, *P < *0.0001; cotton plus α-pinene versus fungus garden plus α-pinene, *P < *0.0001), and 36 h (only α-pinene versus fungus garden plus α-pinene, *P < *0.0001; cotton plus α-pinene versus fungus garden plus α-pinene, *P < *0.0001) compared to most control vials (mixed regression model with time and treatment as fixed effects and ant colony as random effects, α = 0.05). In addition, the 36-h subcolonies had significantly reduced α-pinene compared to the 12-h subcolonies (*P < *0.0001) and the 24-h subcolonies (*P < *0.0001). *L. gongylophorus* strains were tested in a similar fashion. Vials containing *L. gongylophorus* were exposed to α-pinene and the headspace was measured after 36 h of exposure (Fig. 5B). Compared to the control vials, *L. gongylophorus* strains did not reduce α-pinene significantly (*P* = 0.2786, Welch two-sample *t* test).

When subcolonies from five *A. cephalotes* colonies were exposed to linalool (10.6084/m9.figshare.12747029), there was a significant reduction of linalool in the headspace between controls and 12-h subcolonies (only linalool versus fungus garden plus linalool, *P < *0.0001; cotton plus linalool versus fungus garden plus linalool, *P* = 0.0728), 24-h subcolonies (only linalool versus fungus garden plus linalool, *P < *0.0001; cotton plus linalool versus fungus garden plus linalool, *P* = 0.0021), and 36-h subcolonies (only linalool versus fungus garden plus linalool, *P < *0.0001; cotton plus linalool versus fungus garden plus linalool, *P* = 0.0049). We did not see any significant differences between the 12-h, 24-h, and 36-h linalool headspace levels. For the headspace sampling with *L. gongylophorus* (10.6084/m9.figshare.12747029), linalool was significantly reduced (*P* = 0.0036, Welch two-sample *t* test).

## DISCUSSION

Microbes can mediate plant-insect interactions, including influencing a herbivore’s capacity to overcome plant chemical defenses (15). Here, we show PSC detoxification in leaf-cutter ants as a polymicrobial process with bacterial communities that supplement the degradative capabilities of the ants’ fungal mutualist, *L. gongylophorus*. Building on previous literature (46–50), we demonstrate that ant-associated strains of *L. gongylophorus* and *Leucoagaricus* sp. WM170124-07 have variable abilities to tolerate and degrade PSCs (Fig. 2 and 5), suggesting the necessity for additional symbionts to detoxify the diverse defensive compounds that leaf-cutter ants encounter as generalist herbivores. Bacteria we commonly isolate from the fungus gardens of leaf-cutter ants both tolerate and degrade PSCs *in vitro* (Fig. 3 and 4). Whole-genome sequencing of these bacteria revealed the presence of genes involved in PSC degradation (Fig. 3), which is further supported by our analysis of existing fungal garden metagenomes and metatranscriptomes (10.6084/m9.figshare.12747032 and 10.6084/m9.figshare.12747107). Through ant subcolony experiments, using garden substrates with and without PSCs added to the environment, we found *in vivo* degradation of PSCs by fungus gardens (Fig. 5). Taken together, our findings indicate that the symbiotic microbes within the fungus gardens of leaf-cutter ants can detoxify plant defensive compounds, which may contribute to the overall success of these generalist herbivores.

We show that strains of ant-cultivated *L. gongylophorus* and *Leucoagaricus* sp. WM170124-07 vary in their sensitivity to and degradative ability toward different PSCs. For example, in tolerance assays, linalool completely inhibited the growth of almost all *L. gongylophorus* and *Leucoagaricus* sp. strains, while most strains grew unperturbed in the presence of farnesol (Fig. 2). These results fit with the findings of other studies investigating the toxicity of various PSCs to the fungal cultivars of leaf-cutter ants (46–49). Although strains of *L. gongylophorus* appear to be sensitive to many PSCs in petri plate assays, the biosynthetic capacity to degrade some PSCs is present, including the degradation of phenols through production of laccases (50). However, based on genome annotation of *L. gongylophorus* Ac12 and *Leucoagaricus* sp. SymC.cos, the fungal cultivar is unlikely to degrade monoterpenes via known routes of PSC degradation (Fig. 3A and B). In addition to our tolerance assays and *in silico* analyses, we also measured the ability of *L. gongylophorus* to degrade α-pinene or linalool by measuring the headspace of vials containing the fungal mutualist and PSCs. In these experiments, *L. gongylophorus* did not significantly reduce α-pinene (Fig. 5B) but did significantly reduce linalool, which was surprising due to the high inhibition by this compound in the plate assay (Fig. 2B; 10.6084/m9.figshare.12747029). The observed degradation of linalool by *L. gongylophorus* may be due to a difference in dosage, as the tolerance assays contained high concentrations of compound, whereas the headspace sampling had smaller amounts of compound and *L. gongylophorus* obtained a higher biomass (grown for longer on agar plates). Overall, the variation in the ability of *L. gongylophorus* to tolerate and degrade PSCs implies that the bacterial community is potentially involved in reducing PSCs that would otherwise inhibit the fungal mutualist.

Fungus garden bacteria demonstrated high tolerance of PSCs and significant degradation of α-pinene, β-caryophyllene, and linalool. Specifically, we tested bacterial isolates for *in vitro* PSC tolerance and/or degradation, including strains representing the dominant fungus garden microbiome: *Burkholderia*, *Enterobacter*, *Klebsiella*, *Pantoea*, and *Pseudomonas* (27–29, 32). In the tolerance assay, we saw widespread resistance to the PSCs by most bacterial genera, even in the cases of isolates not predicted to contain degradation genes of that PSC’s pathway (Fig. 3A and B). For example, all isolates except for *Acinetobacter* and *Bacillus* were completely resistant to *p*-cymene, perhaps due to an alternative to degradation, such as efflux pumps (51). In addition, while most isolates, except for *Bacillus*, tolerated the eight PSCs, we saw marked differences in their abilities to degrade PSCs. One isolate of *Bacillus*, while inhibited in our tolerance plate assay, significantly reduced α-pinene in our GC-MS experiment (Fig. 4). This could be due to a difference in dosage between the two experiments, which has been shown to have an effect on bacterial tolerance and degradation of PSCs (9). *Burkholderia* and *Pseudomonas*, which both significantly reduced PSCs in our GC-MS assay, have been implicated in PSC degradation in other systems, including bark and mountain pine beetles (8, 52). While not cultured in our study, bacteria in the genera *Serratia* and *Rahnella* isolated from bark beetles were found to degrade PSCs. *Serratia* and *Rahnella* have been detected in leaf-cutter ant fungus garden sequencing studies (32, 33, 36) (10.6084/m9.figshare.12747023), suggesting that other isolates within fungus garden bacterial communities may be involved in degrading PSCs. Finally, no breakdown products were detected via GC-MS, which could be a result of complete degradation of the compounds into components of central metabolism (53, 54).

Garden bacterial genomes and fungus garden metagenomes and metatranscriptomes indicate that genes involved in PSC degradation are present and expressed within the fungus garden bacterial community. Specifically, the presence of cytochrome P450-encoding genes and cumate, *trans*-cinnamate, and *p*-cymene degradation pathways, as well as the presence and *in situ* expression of genes in the α-pinene/limonene and geraniol degradation pathways, indicates that garden bacteria can be predicted to metabolize PSC. In addition, the presence of the gene that encodes squalene-hopene cyclase, which is involved in synthesis of hopanoids and increase of membrane stability (55), in *Asaia* and *Burkholderia* isolates could explain the ability to tolerate stressful conditions, such as growth in the presence of PSCs. With the available data, we were able to predict that both individual garden bacterial isolates and garden bacterial metagenomes contained the genes necessary to degrade or transform PSCs that could harm the fungus gardens. Of note, the higher completeness of pathways observed in the metagenomes suggests that while individual strains may not have the entire pathway for the degradation of a particular compound, as a community, garden bacteria have the capabilities to reduce PSCs within the fungus garden. The concept of facilitation (56), in which one organism accidentally or purposefully receives a benefit from another, in microbial communities has been widely explored, including in the presence of toxins (57). Facilitation between microbes has been observed in the microbiomes of a broad range of organisms (58), such as plants (59), humans (60), and ruminants (61, 62), as well as insects, including in gut microbiomes of honey bees, which contain microbial symbionts that have complementary abilities (63) and where cross-feeding between microbial community members has been observed (64). Further experiments are necessary to address the microbial community potential to complement each other in the degradation of various PSCs and if different community compositions would impact plant intake by the fungus gardens.

We further assessed the capacity of microbes within ant gardens to degrade PSCs by exposing pieces of *A. cephalotes* fungus gardens to α-pinene or linalool and measuring the headspace over time. In the presence of both α-pinene and linalool, the fungus gardens significantly reduced the amount of PSCs in the headspace compared to vials with no fungus garden. After 12 h of exposure, the headspace of fungus gardens contained α-pinene and linalool levels 20% and 52% lower, respectively, than vials containing only PSCs (i.e., no fungus garden). α-Pinene levels in the headspace of fungus garden vials decreased over the 36 h of exposure, whereas linalool levels in the headspace of fungus garden vials remained stable after 12 h, suggesting that there may be a limit to the degradation possible with linalool. Finding significant *in vivo* reduction of PSCs by microbes in the fungus garden, in combination with the *in silico* and *in vitro* evidence of fungal and bacterial PSC tolerance and degradation, indicates the ability of the leaf-cutter ant external digestive system to mitigate the presence of PSCs.

Like other herbivorous insect systems, the gut microbiome of leaf-cutter ants is demonstrably important for dictating palatable plant substrate. Through a combination of *in vitro* and *in vivo* approaches, our study provided evidence that the consistent bacterial community in fungus gardens contributes to the detoxification of PSCs, potentially enabling leaf-cutter ants to forage from a wide variety of plant sources. In addition, previous work has demonstrated that leaf-cutter ants have differing foraging behaviors between species (25, 37, 38), which could possibly be explained by a complexity of factors, including potency of PSCs within different substrates and the fungus garden microbiome’s capacity to degrade these compounds. Overall, the symbioses formed between fungal and bacterial symbionts with leaf-cutter ants demonstrates the intricacy and nuance with which microbes serve as an interface between herbivores and the plants they consume, as well as how microbes contribute to the ecological success of these systems.

## MATERIALS AND METHODS

### *L. gongylophorus* and *Leucoagaricus* sp. WM170124-07 tolerance of PSCs.

We selected compounds for testing based on leaf extracts from plant families that have been foraged by leaf-cutter ants (17, 25, 36–44), detection of terpenes in fungus gardens of *A. laevigata* (32), and commercial availability: 98% (+)-(1*R*)-α-pinene (Acros Organics), >90% β-caryophyllene (TCI), 99% eucalyptol (Sigma-Aldrich), 95% farnesol (Sigma-Aldrich), 96% (−)-(*S*)-limonene (Sigma-Aldrich), 97% linalool [48.2% (−)-(*R*)-linalool/51.8% (+)-(*S*)-linalool] (Sigma-Aldrich), 99+% *p*-cymene (Acros Organics), and 85% terpinolene (Sigma-Aldrich). The eight PSCs were tested against five strains of fungal cultivars from *A. sexdens*, *A. laevigata*, *A. bisphaerica*, *A. capiguara*, and *P. diversus* colonies (isolation information at 10.6084/m9.figshare.12747077). Of note, while *P. diversus* is not within the leaf-cutter ant lineage and largely collects substrates like seeds, insect frass, and dry plant debris for its garden, the species has been observed occasionally collecting fresh leaf and flower material as a substrate for its fungus garden (38, 65). We put a 6-mm fungal plug of *L. gongylophorus* or *Leucoagaricus* sp. WM170124-07 onto a 60-mm Oxoid malt extract agar plate (OMEA; per liter: 30 g of malt extract, 5 g of mycological peptone, 15 g of agar) and cultured for 2 weeks at ambient temperature. Then, for each PSC, we placed a sterile disc with 15 μl of undiluted compound that had been allowed to dry in a biological safety hood for 5 min 1 cm from the edges of fungal growth. We tested each compound in triplicate (three plates per PSC per cultivar), and inhibition was monitored over the course of 2 weeks, with pictures being taken on days 2 and 14. Inhibition was determined by a qualitative scale where 0 represents no inhibition, 1 represents no/slight inhibition at day 2 and normal growth by day 14 (compared to control), 2 represents no/slight inhibition at day 2 and resumption of slow growth by day 14 (compared to control), 3 represents almost complete inhibition by day 2 and no additional growth by day 14, and 4 represents complete inhibition at day 2 and day 14 (Fig. 2A). Inhibition was measured by the same individual based on direct observation.

### Sampling and bacterial isolations.

We collected fungus-farming ant colonies in January 2017 in the following general locations in Brazil: Anavilhanas, Amazonas; Ducke Reserve, Amazonas; Itatiaia, Rio de Janeiro; Botucatu, São Paulo State; and Ribeirão Preto, São Paulo State. Details regarding the exact GPS coordinates and environment of the samples can be found at 10.6084/m9.figshare.12747077. In the field and lab setting, we vortexed pieces of fungus garden from the middle in 1× phosphate-buffered saline (PBS), which we then pipetted onto yeast malt extract agar (YMEA; per liter: 4 g of yeast extract, 10 g of malt extract, 4 g of dextrose, 15 g of agar). Garden bacteria were isolated from multiple fungus gardens of the two genera of leaf-cutter ants, *Atta* and *Acromyrmex*, as well as three genera of other fungus-growing ants, *Paratrachymyrmex*, *Mycetophylax*, and *Apterostigma* (10.6084/m9.figshare.12747077). We obtained pure isolates after several rounds of subculturing based on morphology for a total of 317 isolates. We identified 117 isolates to the genus level by 16S rRNA gene sequencing as previously described (66). Briefly, we lysed colonies and performed PCR with 16S rRNA primers 27F (5′-GAGAGTTTGATCCTGGCTCAG-3′) and 1492R (5′-GGTTACCTTGTTACGACTT-3′). We sequenced samples using Sanger sequencing at the University of Wisconsin—Madison Biotech Center (Madison, WI) and analyzed the sequences using 4Peaks and CLC Sequence Viewer 7. We matched the 16S rRNA gene sequence using BLAST (67) and the SILVA database (https://www.arb-silva.de) for the nearest genus-level identification.

### DNA extraction, assembly, and annotation.

We selected 42 bacterial isolates for whole-genome sequencing, as they belonged to genera known to be abundant and consistent in fungus gardens (*Burkholderia*, *Enterobacter*, *Klebsiella*, *Pantoea*, and *Pseudomonas*) or belonging to genera less common in the fungus garden (*Acinetobacter*, *Asaia*, *Bacillus*, *Chitinophaga*, *Chryseobacterium*, and *Comamonas*). We extracted DNA from 42 bacterial isolates using the Promega Wizard genomic DNA purification kit using the protocol for Gram-negative and Gram-positive bacteria. We used the Qubit BR double-stranded DNA (dsDNA) kit (Invitrogen, USA) for quality control measures. We prepared genomic DNA libraries for Illumina MiSeq 2 × 300-bp paired-end sequencing by the University of Wisconsin—Madison Biotechnology Center. We corrected reads with MUSKETv1.1 (68), merged paired ends with FLASH v1.2.7 (69), and performed assembly with SPAdes 3.11.0 (70). To assess genome assembly quality, we ran a BUSCO analysis (71) in the genome mode (-m genome) with the automated lineage selection (–auto-lineage-prok for bacterial isolates and –auto-lineage-euk for *L. gongylophorus/Leucoagaricus* sp.). Genome statistics for the bacterial isolates can be found at 10.6084/m9.figshare.12747092. We determined species-level identification by uploading the bacterial genomes to JSpeciesWS (72), performing a Tetra correlation search, and taking the first result. If there were conflicts in the top 5 results (i.e., different genera), the top 5 genomes were pulled and average nucleotide identity (ANI) was determined with pyani (73) using ANIm analysis. Then, we selected the genome with the highest percent similarity as our isolate’s taxonomic status. We identified some isolates differently based on whole genomes from the 16S rRNA taxonomic classification, such as one *Burkholderia* isolate (ICBG641) which belonged to the genus *Paraburkholderia* (10.6084/m9.figshare.12747092).

We structurally annotated each genome using Prokka v1.12 (74) and used the amino acid output for functional annotation. We identified predicted proteins putatively involved in PSC degradation from each genome in one of two ways. (i) Using the KEGG Automatic Annotation Server (KAAS), we investigated genes encoding enzymes putatively involved in monoterpene degradation or aromatic compound degradation, as defined in the KEGG limonene and α-pinene degradation pathway (ko00903), geraniol degradation (ko00281), and degradation of aromatic compounds (ko01220, modules 00419, 00539, and 00545). (ii) We used DIAMOND v0.9.21.122 (75) BLASTP against the UniProt, Swiss-Prot and TrEMBL databases (www.uniprot.org/downloads), downloaded on 18 July 2019. We kept only the top 5% of hits (–top 5) that had an E value below 1e−05 for each query sequence. Then, using the grep command, we looked for the following UniProt accession numbers that corresponded to 21 cytochrome P450s (76): P00183, P14779, Q2L6S8, P18326, Q59079, Q59831, Q06069, P53554, P33006, U5U1Z3, A9F9S4, Q59723, Q59990, Q9K498, Q53W59, Q8VQF6, A9FZ85, Q88LH7, Q88LH5, Q88LI2, and Q65A64. We also looked for the following UniProt accession numbers that corresponded to 20 genes in the diterpene degradation cluster (77): Q9X4W9, Q9X4W8, Q9X4X8, Q9X4X7, Q9X4X6, Q9X4X5, Q9X4X4, Q9X4X2, Q9X4X1, Q9X4X0, Q9X4W7, Q9X4W6, Q7BRJ3, Q7BRJ4, Q7BRJ5, Q7BRJ6, Q7BRJ7, Q7BRJ8, Q7BRJ9, and Q9X4X3. Finally, we looked for the UniProt accession numbers corresponding to *saxA* (78), i.e., A0A0N7FW12, and to squalene-hopene cyclase (55), i.e., P33990, P54924, and P33247. We also performed DIAMOND BLASTP using a custom database with solely these gene sequences with a query coverage cutoff of 75% (–query-cover 75) and an E value cutoff of 1e −05. If there was alignment in both the UniProt analysis and the custom analysis, then genes were predicted to be present. Due to genome fragmentation, predicted genes were only labeled as absent (0) or present (1); copy numbers were not considered. The annotation methods were used on individual bacterial genomes, one *L. gongylophorus* genome (BioProject no. PRJNA179280), one *Leucoagaricus* sp. genome (BioProject no. PRJNA295288), and 12 publicly available leaf-cutter ant garden bacteria metagenomes from Brazil (Gold Analysis Project identifiers Ga0157357 to Ga0157368). For the metagenomes, we used the metagenomes KAAS option instead of the complete/draft genome KAAS option.

### Bacterial tolerance of PSCs.

We tested the effects of the eight PSCs on 46 bacterial isolates using Whatman 6-mm discs. Bacterial isolates were grown overnight (16 to 24 h) until turbid (optical density at 600 nm [OD_600_] of ∼1 to 2). We spread 100 μl of overnight culture on YMEA plates using a glass cell spreader. We deposited a disc with 15 μl of PSC in the center of the bacterial lawn. Each PSC was tested in triplicate (3 plates per PSC per bacterial isolate). After 48 h, we took pictures of the plates using an Epson scanner and then uploaded the photos into Fiji (79). We used Fiji v1.0 to measure the zones of inhibition caused by each PSC (in centimeters). We calculated the average of the three zones of inhibition and then scaled all the zones of inhibition in reference to the largest zone observed so that 0 indicates inhibition (zone of inhibition = 3 cm) and 1 indicates no inhibition (100% growth; zone of inhibition = 0 cm).

### Phylogenetic trees.

We generated a genome-based, multilocus fungus garden bacterial phylogeny based on previous methods (80). Briefly, we used 93 full TIGRFAM proteins in the “core bacterial protein” set (GenProp0799) as the molecular data set. We aligned the protein sequences with the top HMMer bit score for each protein family using MAFFT (81), which we then converted to codon alignments and concatenated. We used RAxML-7.2.6 (82) to generate phylogeny using the GTRgamma substitution model and 100 rapid bootstraps on the final, recombination-free alignment. We generated the gene tree-based phylogeny using ASTRAL-II. The code for this process can be found at https://github.com/chevrm/core_species_tree. Phylogenies were visualized and edited in FigTree v1.4.3 (83).

### Metatranscriptomic sequencing of fungus gardens.

We collected samples directly from the field into RNAlater buffer. We took samples from the top sections of three different colonies: two *A. cephalotes* colonies from La Selva Biological Station, Costa Rica, and one *A. colombica* colony from Golfito, Costa Rica (10.6084/m9.figshare.12747107). Total RNA extraction was identical to a method previously described (84). We performed cDNA library construction and Illumina HiSeq2000 sequencing at the University of Wisconsin Biotechnology Center (Madison, WI). We uploaded the metatranscriptomes to MG-RAST and processed the reads with their standard operating procedure (85). We downloaded the reads postprocessing (quality reads) and then analyzed metatranscriptomes using prodigal v2.6.2 (86), DIAMOND v0.9.21.122, and kallisto v0.43.1 (87). First, we ran prodigal on the assembled nucleotide files of 12 garden bacterial metagenomes from Brazil (downloaded from JGI; accession numbers provided in annotation methods above) with the metagenomic flag (-p meta). Then, we created a kallisto index with all of the combined prodigal garden bacterial metagenome output. We used the kallisto quant command to pseudoalign the garden bacterial index against the metatranscriptome reads. This gave a transcripts per million (TPM) value of bacterial transcripts in the metatranscriptome. Then, we used DIAMOND to BLASTp search the metagenome coding regions against the UniProt and KEGG databases described above. Using grep, we found the genes of interest (same as in the bacterial isolate annotation) and connected the gene of interest, the metagenomic transcript it mapped to, and the TPM in the metatranscriptome. For genes with multiple transcripts and different TPMs, we recorded the unique values (10.6084/m9.figshare.12747032) and summed the TPMs for total expression (10.6084/m9.figshare.12747113). We also did the same workflow for four housekeeping genes (*gyrB* [K02470], *rpoB* [K03043], *rpoD/sigA* [K02086], and *rpsL* [K02950]).

### GC-MS of bacterial isolates incubated with PSCs.

We prepared 15 bacterial isolates representing highly resistant genera (*Burkholderia*, *Enterobacter*, *Klebsiella*, *Pantoea*, and *Pseudomonas*) and inhibited genera (*Bacillus*) in one of two ways for gas chromatography-mass spectrometry (GC-MS): addition of compound during exponential growth and addition during stationary growth. For both methods, bacterial isolates were grown overnight (16 to 24 h) in 10% tryptic soy broth (TSB). All shaking was done at 300 rpm and room temperature. All experiments included an extra vial to read the OD_600_ to ensure bacterial growth in the presence of compound, which also served to confirm earlier patterns of compound tolerance by bacterial isolates (Fig. 3C; 10.6084/m9.figshare.12747035).

### (i) Exponential phase.

We diluted overnight cultures to an OD_600_ of 0.08. We inoculated the appropriate amount of overnight culture into vials containing 10% TSB and 2.5 μl/ml of one of four PSCs, α-pinene, β-caryophyllene, eucalyptol, and linalool, that were added using a glass manual GC syringe (10 μl; Thermo Scientific). We left the bacterial cells and plant compound shaking for another 2 days at room temperature. This was done with 15 bacterial isolates representing six genera, as well as negative controls (no bacteria), in triplicate (16 × 4 compounds × 3 replicates).

### (ii) Stationary phase.

We pipetted 10 μl of overnight culture into 987.5 μl of 10% TSB in vials. After 2 days of incubation at room temperature with shaking at 300 rpm, we added 2.5 μl/ml of one of four PSCs (α-pinene, β-caryophyllene, eucalyptol, and linalool) directly to the vials using a glass manual GC syringe (10 μl; Thermo Scientific). Then, we left the bacterial cells and plant compound shaking for another 2 days at room temperature. This was done with the same number of samples as listed above.

For both methods i and ii, we extracted PSCs by pipetting 1 ml of hexane into each vial and shaking the vials overnight. We removed 500 μl of the hexane-PSC phase and put into new vials containing 500 μl of hexane and 5 μl/ml of the internal standard toluene. We then analyzed the abundance of each PSC using GC-MS. Specifically, the GC system consisted of a Thermo Fisher Trace 1310 gas chromatograph coupled with Thermo ISQ LT single-quadrupole spectrometer. We injected 1 μl of each mono-/sesqui-terpene sample directly, with a split flow ratio of 30:1. We used an oven profile of 40°C, followed by a ramp of 3°C min^−1^ to 115°C (monoterpenes) or 130°C (sesquiterpenes) and then 30°C min^−1^ to 250°C with a 2-min hold. We integrated and analyzed peaks using the Chromeoleon chromatography data system software.

We integrated and standardized signal peaks from the GC based on the internal standard toluene (peak area/internal standard peak area) for each vial. In addition, we used standard curves of the four pure PSCs to measure changes in concentration in the samples compared to controls. We made standard curves to incorporate the possible ranges of concentrations (0 μl/ml to 3.5 μl/ml) within the experiment. We then calculated proportional change of bacterial treatments versus the nonbacterial control. Specifically, we took the average of the nonbacterial control standardized peak areas and subtracted the control average from all the bacterium-compound peak areas. Then, we divided the adjusted value by the nonbacterial control average to obtain the percent change [(bacterial standardized peak area – average of control standardized peak areas)/average of control standardized peak area]. We then analyzed the standardized values in JMP Pro 13 by performing one-sample Student’s *t* tests for each compound with a null hypothesis of μ = 0, representing no change between compound abundance in bacterium-treated and the nonbacterial control. Since we were performing 15 separate statistical tests for each compound (between nonbacterial control and each of the 15 bacterial isolates), we used a Bonferroni correction to avoid false positives (α = 0.05/15 = 0.0033).

### Headspace sampling of fungus garden subcolonies with PSCs.

*Atta cephalotes* colonies (10.6084/m9.figshare.12747095) that have been maintained in lab since 2012–2018 were used in this experiment to create subcolonies. 16S rRNA gene amplicon sequencing was used to confirm that the bacterial genera in these fungus gardens were consistent with isolates used throughout this study (10.6084/m9.figshare.12747119, 10.6084/m9.figshare.12747023, and 10.6084/m9.figshare.12747068). We prepared 20-ml, 18-mm Restek (Bellefonte, PA) vials (catalog number 23082) with magnetic screw-thread caps (catalog number 23090) three ways with α-pinene or linalool: (i) empty vials with a 20-μl Accu-Fill 90 micropipette cut to 2.54 cm and flame-sealed at one end containing 1 μl of PSC (*n* = 6), (ii) vials with approximately 0.3 to 0.4 g of cotton with a 2.54-cm micropipette flame-sealed at one end containing 1 μl of PSC (*n* = 3), and (iii) vials with approximately 0.3 g of fungus garden material with all ants manually removed and a 2.54-cm micropipette flame-sealed at one end containing 1 μl of PSC (*n* = 3 subsamples × 3 to 5 different *A. cephalotes* colonies). We used material from three colonies in the α-pinene experiment and five colonies in the linalool experiment. We also prepared samples of vials with only fungus garden (i.e., no exposure to PSC) during certain runs to ensure that there were no detectable PSCs innate to the system. This was done for three separate time points based on exposure to a PSC: 12 h postexposure, 24 h postexposure, and 36 h postexposure. At these time points, we destructively sampled each respective set of vials with a Shimadzu HS20 headspace sampler coupled to a Shimadzu GC-2010 Plus instrument with a flame ionization detector. Specifically, we loaded vials into the headspace sampler and injected into a column with a 50:1 split flow ratio. For the vials with α-pinene, the headspace sampler and oven were at 60°C, followed by a 20°C/min ramp up to 140°C. For the vials with linalool, which has a higher boiling point than α-pinene, the headspace sampler and oven were kept at 70°C, followed by a 25°C/min ramp up to 205°C. Then, we identified compounds using retention time (α-pinene = 3.2 min; linalool = 6.2 min) and calculated areas under the curve in Shimadzu’s LabSolutions software to determine the relative difference in α-pinene or linalool between vials. We chose α-pinene and linalool because they are compounds that the garden bacteria can degrade and are inhibitory against *L. gongylophorus* and *Leucoagaricus* sp. WM170124-07.

Since we took subsamples (subcolonies) from each *A. cephalotes* colony (3 subsamples × 3 time points × 5 colonies), we employed a linear mixed-effects model to account for the correlation (nonindependence) between subsamples. Specifically, to test if ant colony had an effect on the observed value, we used lmer package v3.1 to 0, holding time and treatment as the fixed effects and ant colony as the random effect. Before the analysis, we divided the values by 1,000,000 to rescale the response for the lmer optimization procedure. For the α-pinene treatment, the colony variance is reported as 0, indicating that the variability with respect to ant colony is much smaller than the variability with respect to the residual error. For the linalool treatment, the colony variance was 0.000226, indicating that some of the variability observed was due to the sampling from different colonies. We then used the estimated marginal means (EMMs) with the emmeans package v1.3.5 for linear regression analysis of the data, using the pairs() method. Marginal means were compared pairwise between exhaustive two-way level combinations of treatment (control, cotton, and fungus garden) and of time (12 h, 24 h, and 36 h). Assumptions of normality, linearity, and homoscedasticity for linear regression were examined by plot diagnostics and were met for each analysis. All the code used in this analysis is available at github.com/cfrancoeur/PSC.

### Headspace sampling of *L. gongylophorus*.

We isolated *L. gongylophorus* strains by plating small pieces of healthy fungus garden from laboratory colonies on potato-dextrose agar (PDA). Laboratory fungus-farming ant colonies are kept in a temperature-controlled (28°C) room in separate large plastic containers. We used five *A. cephalotes* colonies collected over the course of several years (2012 to 2018) from Costa Rica (10.6084/m9.figshare.12747095). In addition, several isolates from Brazilian *Atta* gardens used in the fungal cultivar tolerance experiment (Fig. 2) were included.

We pipetted 2 ml of PDA into 20-ml, 18-mm Restek vials with magnetic screw-thread caps and left it to solidify on a slant. Then, 3- by 3-mm pieces of freshly growing *L. gongylophorus* strains were placed onto the slant and grown for 1 month at room temperature in the dark. We prepared three vials for each of the *A. cephalotes* cultivars (*n* = 5 strains × 3 vials × 2 compounds), and we prepared one vial for three additional *L. gongylophorus* strains: AB1, AL2, and AS1 (*n* = 3 strains × 1 vial × 2 compounds). After the month of growth, we filled 20-μl Accu-Fill 90 micropipettes (Becton, Dickinson and Company, NJ) cut to 2.54 cm and flame-sealed at one end with (i) nothing, (ii) 1 μl of α-pinene, or (iii) 1 μl of linalool. We then put the filled micropipettes into the vials and after 36 h of exposure, we analyzed the headspace of the vials with the same methodology described for the subcolony headspace sampling. We statistically compared signal peaks using a Welch two-sample *t* test, comparing the peaks from the control vials to those from the vials containing *L. gongylophorus*.

### Data availability.

All sequencing data have been uploaded to NCBI under the following BioProject numbers: PRJNA564151, PRJNA429666, PRJNA429667, PRJNA429668, PRJNA565936, PRJNA577467. Individual accession numbers for each data set can be found at 10.6084/m9.figshare.12747092 and 10.6084/m9.figshare.12747107. Supplemental data can be found at https://figshare.com/projects/mBio_Francoeur/86891.
