# Erratum for Francoeur et al., “Bacteria Contribute to Plant Secondary Compound Degradation in a Generalist Herbivore System”

**DOI:** 10.1128/mBio.01827-21

**Published:** 2021-08-03

**Authors:** Charlotte B. Francoeur, Lily Khadempour, Rolando D. Moreira-Soto, Kirsten Gotting, Adam J. Book, Adrián A. Pinto-Tomas, Ken Keefover-Ring, Cameron R. Currie

**Affiliations:** a Department of Bacteriology, University of Wisconsin—Madison, Madison, Wisconsin, USA; b Department of Energy Great Lakes Bioenergy Research Center, University of Wisconsin—Madison, Madison, Wisconsin, USA; c Sección de Entomología Medica, Departamento de Parasitología, Facultad de Microbiología, Universidad de Costa Rica, San José, Costa Rica; d Laboratory of Genetics, University of Wisconsin—Madison, Madison, Wisconsin, USA; e Centro de Investigación en Estructuras Microscópicas, Universidad de Costa Rica, San José, Costa Rica; f Departamento de Bioquímica, Facultad de Medicina, Universidad de Costa Rica, San José, Costa Rica; g Centro de Investigación en Biología Celular y Molecular, Universidad de Costa Rica, San José, Costa Rica; h Departments of Botany and Geography, University of Wisconsin—Madison, Madison, Wisconsin, USA

## ERRATUM

Volume 11, no. 5, e02146-20, 2020, https://doi.org/10.1128/mBio.02146-20. We left out the appropriate permit information for collections done in Brazil. The following line should have been added to Acknowledgments (PDF page 16): “In Brazil, collections of biological samples and research on genetic resources were authorized by SISBIO permits 46555-1 to 46555-7 and CNPq permit 010936/2014-9.” This change does not affect the main text of the article in any way.

